# Deep learning models for webcam eye tracking in online experiments

**DOI:** 10.3758/s13428-023-02190-6

**Published:** 2023-08-22

**Authors:** Shreshth Saxena, Lauren K. Fink, Elke B. Lange

**Affiliations:** 1 Music Depart., Max Planck Institute for Empirical Aesthetics, Frankfurt am Main, Germany; 2 Dept. of Psychology, Neuroscience & Behaviour, McMaster University, Hamilton, Ontario Canada; 3 Max Planck - NYU Center for Language Music & Emotion, Frankfurt am Main, Germany

**Keywords:** Online, Low resolution, Eye tracking, Deep learning, Computer vision, Eye gaze, Fixation, Free viewing, Smooth pursuit, Blinks

## Abstract

Eye tracking is prevalent in scientific and commercial applications. Recent computer vision and deep learning methods enable eye tracking with off-the-shelf webcams and reduce dependence on expensive, restrictive hardware. However, such deep learning methods have not yet been applied and evaluated for remote, online psychological experiments. In this study, we tackle critical challenges faced in remote eye tracking setups and systematically evaluate appearance-based deep learning methods of gaze tracking and blink detection. From their own homes and laptops, 65 participants performed a battery of eye tracking tasks including (i) fixation, (ii) zone classification, (iii) free viewing, (iv) smooth pursuit, and (v) blink detection. Webcam recordings of the participants performing these tasks were processed offline through appearance-based models of gaze and blink detection. The task battery required different eye movements that characterized gaze and blink prediction accuracy over a comprehensive list of measures. We find the best gaze accuracy to be 2.4° and precision of 0.47°, which outperforms previous online eye tracking studies and reduces the gap between laboratory-based and online eye tracking performance. We release the experiment template, recorded data, and analysis code with the motivation to escalate affordable, accessible, and scalable eye tracking that has the potential to accelerate research in the fields of psychological science, cognitive neuroscience, user experience design, and human–computer interfaces.

**Supplementary Information:**

The online version contains supplementary material available at 10.3758/s13428-023-02190-6.

## Introduction

Eye movements offer direct insight into the dominant visual stream of attention and have been widely studied across disciplines of cognitive neuroscience and psychology to understand human behavior, perception, and attention (Radach et al., 2003). The diverse set of events that can be recorded from the eye, such as fixations, saccades, smooth pursuit, and blinks, make eye tracking a valuable method for researchers and practitioners. However, though eye tracking methods have a long history of development and application (Buswell, 1935; Delabarre, 1898; Huey, 1898; Yarbus, 1967), popular recording methods to measure ocular activity mostly rely on expensive, specialized, and delicate hardware that limits the application of eye tracking to highly controlled and unnaturalistic setups. Such hardware-based eye trackers (e.g., EyeLink 1000 by SR Research; iView X systems by SensoMotoric Instruments) provide high spatial and temporal resolution of recorded data but impose limitations on the recording environment, user movements, and scalability of data collection. Development of portable hardware in the form of wearable glasses or head-mounted devices (e.g., Pupil Core headset; Tobii Pro glasses; UltraFlex headgear by Positive Science) aids in improving affordability, adaptability, and non-intrusiveness of eye trackers, however they have seen limited adoption to large-scale "in-the-wild" applications. Additionally, the majority of these methods are developed as proprietary closed-source solutions, limiting user-driven development, customization and extension of offered methods, extensive performance evaluation, and compatibility of vendor products with external hardware or software.

Software-based approaches, applying sophisticated computational algorithms, have demonstrated the ability to track eye movements using general-purpose, consumer-grade, off-the-shelf cameras in unrestricted environments (Hansen & Pece, 2005; Papoutsaki et al., 2016; Krafka et al., 2016; Zhang et al., 2015; Xu et al., 2015; Zhang et al., 2019; Kellnhofer et al., 2019). Such methods greatly reduce dependence on specialized hardware and allow highly affordable, large-scale applications of eye tracking. These software-based approaches can be broadly classified into model-based and appearance-based methods. Model-based methods (see Hansen & Ji, 2010, for an overview) estimate geometric eye shapes or 2D features, such as pupil-center, position of corneal reflection, eye corners, iris contours, etc., from images of the eye typically captured using infrared light sources. These features are used to fit a person-specific, 3D model of the eye to detect gaze or to directly estimate gaze using regression. Due to their rigid geometric assumptions, model-based methods are highly sensitive to lighting or illumination changes, head movements, user distance, intrinsic and extrinsic camera parameters, and preset thresholds. Appearance-based methods (see Cheng et al., 2021, for an overview), on the other hand, rely on high-level visual features extracted from RGB images of the face and/or eyes to predict outputs such as gaze angle, point of gaze, and blink probability using robust machine learning models. Appearance-based methods have lately surpassed model-based methods for in-the-wild applications with low-resolution images and are more robust to changing environments in less restricted setups (Zhang et al., 2019, provide a detailed comparison).

One potential application for appearance-based eye tracking is in online, webcam-based scientific experiments or user research. Online studies incorporating eye tracking enable researchers to sample global populations in shorter time frames and to replicate results cross-culturally, potentially improving the ecological and external validity of research. Nonetheless, the task of tracking eye movements from a limited resolution webcam image in an online experiment setting is challenging, given the high variability in participant appearance, environmental factors (lighting, reflection, etc.), and hardware specifications (screen size, camera resolution, etc.). Video artefacts, such as motion blur, and unrestricted movement parameters, such as head pose, further add to this challenge. Existing online webcam-based gaze-tracking methods apply real-time image processing and computer vision to learn a regression function from observed facial features, such as pupil coordinates or eye image pixels, to predict gaze points on the screen (Papoutsaki et al., 2016; realeye.io; xlabsgaze.github.io; Xu et al., 2015). These methods make online eye tracking accessible and convenient; however, their robustness to diverse eye-movement types, environments, subjects, and long trial lengths is limited. Previous studies have reported a gaze accuracy between 3 and 4° using the best performing WebGazer model (Papoutsaki et al., 2016; Semmelmann & Weigelt, 2018).

Here, for the first time, we applied appearance-based deep learning methods for eye tracking to webcam videos recorded during an online experiment. We characterized the performance of these methods using a battery of eye tracking tasks: *fixation, zone classification, smooth pursuit, free viewing*, and *blink detection*. The battery provided an extensive benchmarking of eye-tracker performance across different types of ocular movements and allowed comparison with laboratory-based eye tracking from EyeLink 1000 and Pupil Core, evaluated on the same tasks by other researchers (Ehinger et al., 2019). We used three of the best-performing gaze-estimation models: MPIIGaze (Zhang et al., 2015), ETHXGaze (Zhang et al., 2020), and FAZE (Park et al., 2019); and two blink detection algorithms: EAR (Soukupová & Cech, 2016) and RT-BENE (Cortacero et al., 2019), to analyze the data collected in our online study. We split the online data collection and offline model inference steps, reducing the computational restriction of real-time browser inference and allowing flexible, within-subject, comparison of multiple models.

## Method

The study procedure was pre-registered prior to any human observation, and prior to the full collection of data, together with an addendum specifying updates on defining criteria to exclude noisy recordings (Saxena et al., 2021). All ethical approvals, participation criteria, and general procedures can be found in more detail there. We designed and systematically compared calibration strategies to estimate physical parameters and improve gaze prediction accuracy, in our previous publication (Saxena et al., 2022), using a subset of data from the current experiment. The current paper utilizes the best performing calibration strategy from our previous results and reports calibration results on the final dataset in Supplementary Information.

### Participants

Participants in our experiment were recruited through a database of community volunteers of the Max Planck Institute for Empirical Aesthetics, the institute website, and social media channels. In total, 118 participants completed the online study. Data for 53 participants had to be excluded based on inconsistent recording frame rates, face detection failures, and missing files (for a detailed report on unexpected data attrition and updated exclusion criteria, see “Addendum to OSF_Prereg_WebET.pdf” in Saxena et al., 2021). The final dataset consisted of 65 participants (20 male and 45 female), aged 20 to 35 years (M = 26, SD = 3). Forty-six of the participants reported having normal vision and 19 reported corrected-to-normal vision. Fifty-six identified as students. Prior to the study, participants confirmed they met the following requirements: aged between 18 and 35 years (inclusive); not wearing glasses; normal hearing ability; not prone to epilepsy or migraine; not suffering from any neurological disease; slept longer than 5 h the previous night; not under the influence of drugs or alcohol (in the past 72 h). Participants took around 48 min (IQR: 41.75–52.67 min) to complete the study and were reimbursed 14€, with an additional 7€ per half hour if they took more than 60 min.

### Apparatus

Stimulus presentation and data (webcam video and participant responses) recording for our study were done online on the LabVanced (Goeke et al., 2017) platform. All tasks were performed in full-screen mode and were designed to be presented in a fixed coordinate system of frame units (FU) where 1° (visual degree) = 54.05 FU (for estimation of visual angles see Device Calibration in Saxena et al., 2022, and the Procedure section below). All stimuli were presented within a window size of 29.6° × 16.65° (1600 × 900 FU), centered on the screen for all screen sizes. A laptop screen equal to or larger than this size was therefore required for participating. During the experiment, events were recorded as UNIX timestamps and participant responses during tasks were collected using the left-click button. The online experiment was preloaded on the participant’s device and all recordings were transferred only at the end of the experiment to ensure precise stimulus timing and no data loss during transfers. Further, the online experiment tested runtime execution time at regular intervals (every 5 s). We used the mean and standard deviation of recorded execution times of these functions to ensure a working network bandwidth.

### Eye tracking task battery

To evaluate eye tracking recordings and benchmark model performance, we build upon a published battery of eye tracking tasks (Ehinger et al., 2019), selecting the *fixation, free viewing, smooth pursuit,* and *blink detection tasks*, which could be executed with the temporal and spatial resolution of the current webcam methods. We complement the battery with a new *zone classification* *task*, which resembles task settings requiring less precise fixations in a region-of-interest.

#### Fixation

**Fig. 1:**
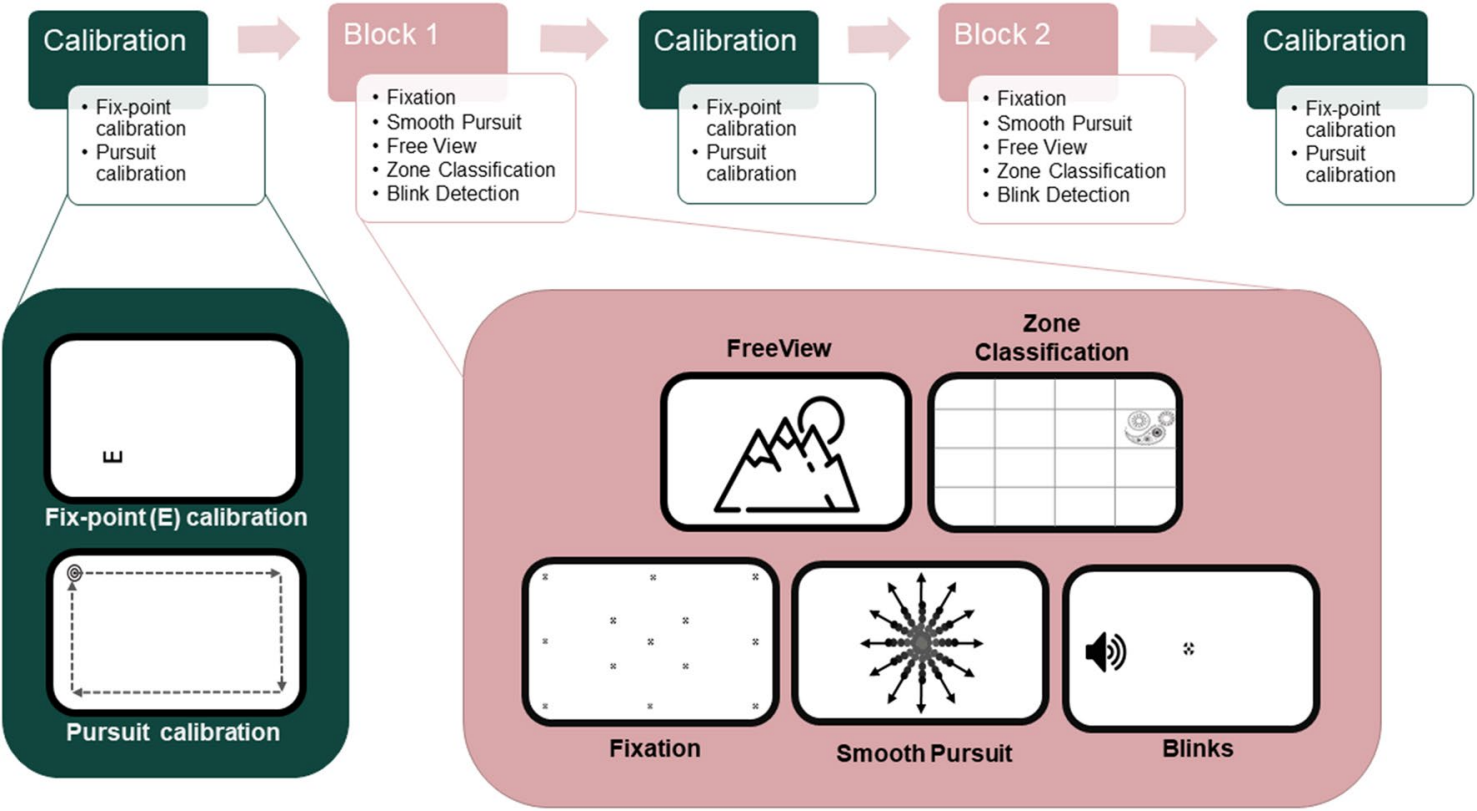
Overview of the experimental procedure and tasks. Task blocks (*pink*) were book-ended by calibration blocks (*green*). The timeline in the figure represents one possible sequence of tasks

The *fixation task* was implemented similar to Ehinger et al.’s, 2019, small-grid task. The fixation target was presented at one of 13 screen locations, with the serial order of locations randomized for each trial and participant. The locations were a subset from locations within a 7 × 7 grid, equally spaced in a range from – 6.2° to 6.2° (visual degrees) vertically and – 11.1° to 11.1° horizontally (see Fig. 1, showing all 13 locations). The fixation target used was a combination of bullseye and cross-hair (Ehinger et al., 2019) with the diameter fixed to 0.6°. Participants were instructed to respond when they fixated on the displayed target and to keep fixating until the target moved to a new location, and repeat. The target moved after a fixed duration of 2500 ms, starting and ending in the center position for each block of trials. Note that the *fixation task* differed from our *fix-point calibration task* in our Gaze Calibration procedure in several aspects, such as the fixation target, the requirement of a visual discrimination task, and the presentation locations of the fixation target on the screen (see *fix-point calibration* in Saxena et al., 2022).

#### Zone classification

We split the presentation screen into a non-visible 4 × 4 grid of equally sized units (7.4° × 4.2°). In each trial, a rangoli pattern appeared for 1500 ms sequentially in one of all 16 grid locations, with the serial order of locations randomized, and a 200-ms gap between presentation offset and onset at a different location. The rangoli pattern was monochrome with multiple centers to motivate different fixational target locations within the pattern (see Fig. 1; different from Fig. 1, the grid was not visible). Participants were instructed to look at the displayed pattern such that their gaze was constrained within its outer boundary.

#### Free viewing

The *free-viewing* *task *presented a subset of 16 images from a publicly available dataset (Judd et al., 2009) to each participant in a randomized order. Each trial presented a single image and participants were instructed to freely explore these images. All images were scaled to maximum presentation size (29.6° × 16.65°), saving the aspect ratio, and displayed for a duration of 3 s each, followed by a fixation cross for 1 s between every two images.

#### Smooth pursuit

The *smooth pursuit task* was an adaptation of the step ramp paradigm (Ehinger et al., 2019; Liston & Stone, 2014). The target for this task was a bullseye with a black outer circle of diameter 0.5° and a white inner circle of diameter 0.25°. Participants were instructed to fixate on the target located in the center of the screen and initiate the movement of the target, which started with a random delay sampled from an exponential distribution with mean 0.5 s, a constant offset of 0.2 s, and truncated at 5 s. The target moved linearly at a speed of 15°/s and the direction of movement was chosen randomly without replacement from one of 12 possible angles spread evenly across the center of a unit circle (see Fig. 1, depicting all possible angles; none of the directions was visualized on the screen but only the target was visible). The starting point for each movement was chosen such that it took 0.2 s for the target to move from the starting point to the center, minimizing the chance of catch-up saccades. The movement ended when the target reached a distance of 8.3° from the center. Participants were instructed to closely follow the target movement with their gaze.

#### Blink detection

The *blink detection task* was implemented as in Ehinger et al., 2019. Participants were instructed to fixate on a central fixation target (same as *fixation task*) and blink each time they heard a beep sound. The beep sound was a 100-ms 300-Hz sine wave apodized (fade in/out) at the beginning and end, generated using the ThinkDSP (Downey, 2013/2022) Python library. Each trial of the task consisted of seven beeps with a pause of 1.4 s between beeps, uniformly jittered by ± 0.2 s to make beeps less predictable to participants.

### Procedure

The online experiment began by providing a description of the study objectives and collecting participants’ consent and general information. This was followed by a Device Calibration procedure, which was important to estimate physical screen–camera–participant relationships. The Device Calibration consisted of two tasks. Participants’ screen size and resolution were estimated by placing an ID card onto the screen and adjusting a reference image to the standardized measure of the card. The distance between participant and screen was controlled by estimating the location of the blind spot in the eye, given a required sitting distance of 50 cm (± 3.5 cm). Physical parameters calculated from these two tasks allowed presenting consistent stimuli on different screen sizes and reinforced participants’ viewing distance for gaze predictions (see Saxena et al., 2022, for a detailed description). Then the task blocks followed, consisting of repeated Gaze Calibration and the tasks from our eye tracking battery (see Fig. 1 for an overview). Gaze Calibration was realized by a 16-pt fixation task, as well as a rectangular smooth pursuit task (*fix-point calibration* and *pursuit calibration*; see Saxena et al., 2022 for a detailed description; see Saxena et al., 2022 and Supplementary Information provided in this paper for comparisons of the efficiency of different calibration strategies). Together, Device Calibration and Gaze Calibration took about 3 min (median time spent for a single trial of fix-point calibration: 14.39 s, and smooth pursuit calibration: 26.35 s).

Each task in the battery was performed for ten trials except for the *free-viewing* *task* which consisted of 16 trials (one for each image) in total. The trials for each task were equally split into two identical blocks, bookended by the two Gaze Calibration tasks. The first block included one additional example trial for all tasks. The sequence of tasks inside the blocks was balanced with a Latin square design. The sequence of two calibration tasks was randomized every time for each block. An example series of tasks one participant might experience during the experiment is presented in Fig. 1.

### Gaze and blink detection models

To evaluate the performance of more sophisticated deep learning architectures for online experimentation we selected three models: the LeNet-based MPIIGaze (Zhang et al., 2015), which was among the first-ever models to apply deep learning for gaze estimation, the FAZE (Park et al., 2019), which utilizes meta-learning to train an adaptable gaze estimator, and ETHXGaze (Zhang et al., 2020) that was trained on a high-quality large-scale dataset collected under extreme head pose and gaze variation. In addition to the gaze estimation models, we selected two blink estimation methods termed as RT-BENE and EAR for detecting blinks from webcam recordings. RT-BENE (Cortacero et al., 2019) uses a trained, end-to-end, convolutional neural network (CNN) that takes the left and right eye images as input and outputs a blink probability score. The network is trained on a custom dataset with over 10,000 blink instances. In contrast, EAR (Soukupová & Cech, 2016) detects blinks using a simple measure called “Eye Aspect Ratio”, which represents the portion of the eye visible in a frame, calculated as pixel distances of eye landmarks. The EAR method leverages robust accuracy of deep-learning-based facial landmark estimators and classifies blinks in real-time.

We applied the models from their open-source implementations, with default image processing and data normalization steps. Face detection models used to extract facial keypoints for MPIIGaze, ETHXGaze, and EAR were updated from the default histogram of gradients method in Dlib (King, 2009) to the more accurate and robust max-margin (MMOD) CNN face detector in Dlib (King, 2015). The MMOD model resulted in quicker inference due to Graphics Processing Unit (GPU) compatibility and could detect faces under varying head poses, lighting conditions, and occlusion, allowing inclusion of four additional participants. All analysis code used to classify eye movements and compute task measures defined below is made available publicly (see Data & Code Availability).

### Data treatment

Data exclusion was performed based on incomplete data recordings and missing data due to inconsistent frame rates or face detection failures (see “Addendum to OSF_Prereg_WebET.pdf” in Saxena et al., 2021, for further description). The final dataset consisted of webcam recordings with a mean frame rate of 29.9 frames per second (fps) (SD = 0.66, SEM = 0.08) that were processed offline through the gaze estimation and blink detection models.

Prior to model inference, all videos were resized to a resolution of 640*480 using OpenCV’s (Bradski, 2008) inter-area interpolation. Resized frames were fed sequentially to each model, generating time series of predictions. Timestamps recorded during the experiment were mapped to video frame numbers using a constant fps, calculated from the recorded meta data, and used to mark events in the predicted time series. All spatial distances were calculated in FU and converted to visual degree (see Apparatus). For aggregating task measures over all trials and participants we use 20% winsorized means, as in Ehinger et al., 2019 (i.e., setting data below the 10th percentile to the 10th percentile, and data above the 90th percentile to the 90th percentile). While this is the proper way to ensure a fair comparison with their results, we do realize that winsorizing shapes the data in favor of accuracy.

Event detection for eye tracking with 30-Hz resolution is not well established and cannot directly borrow from the traditional velocity- and density-based approaches (Andersson et al., 2010; Salvucci & Goldberg, 2000; Shic et al., 2008). We therefore extract eye movements for *fixation, smooth pursuit,* and *zone classification tasks* by time locking gaze predictions around the respective target movement presented to the participants. For the *free-viewing* and *blink detection* *tasks*, we propose suitable event detection methods that can be applied to data with 30-Hz resolution.

To compare model performance across tasks we select one accuracy measure from each task (see Model comparisons across tasks). Accuracy from the *fixation* *task* and angular deviation from *smooth pursuit task* were log-transformed to satisfy approximate normality and have similar variations to the other task scores. Classification accuracy from *zone classification* *task* and the average AUC score from *smooth pursuit* *task* were inverted to have similar order as other scores, i.e., lower magnitude is better. All task scores were z-scored.

### Task measures

#### Fixation

**Fig. 2:**
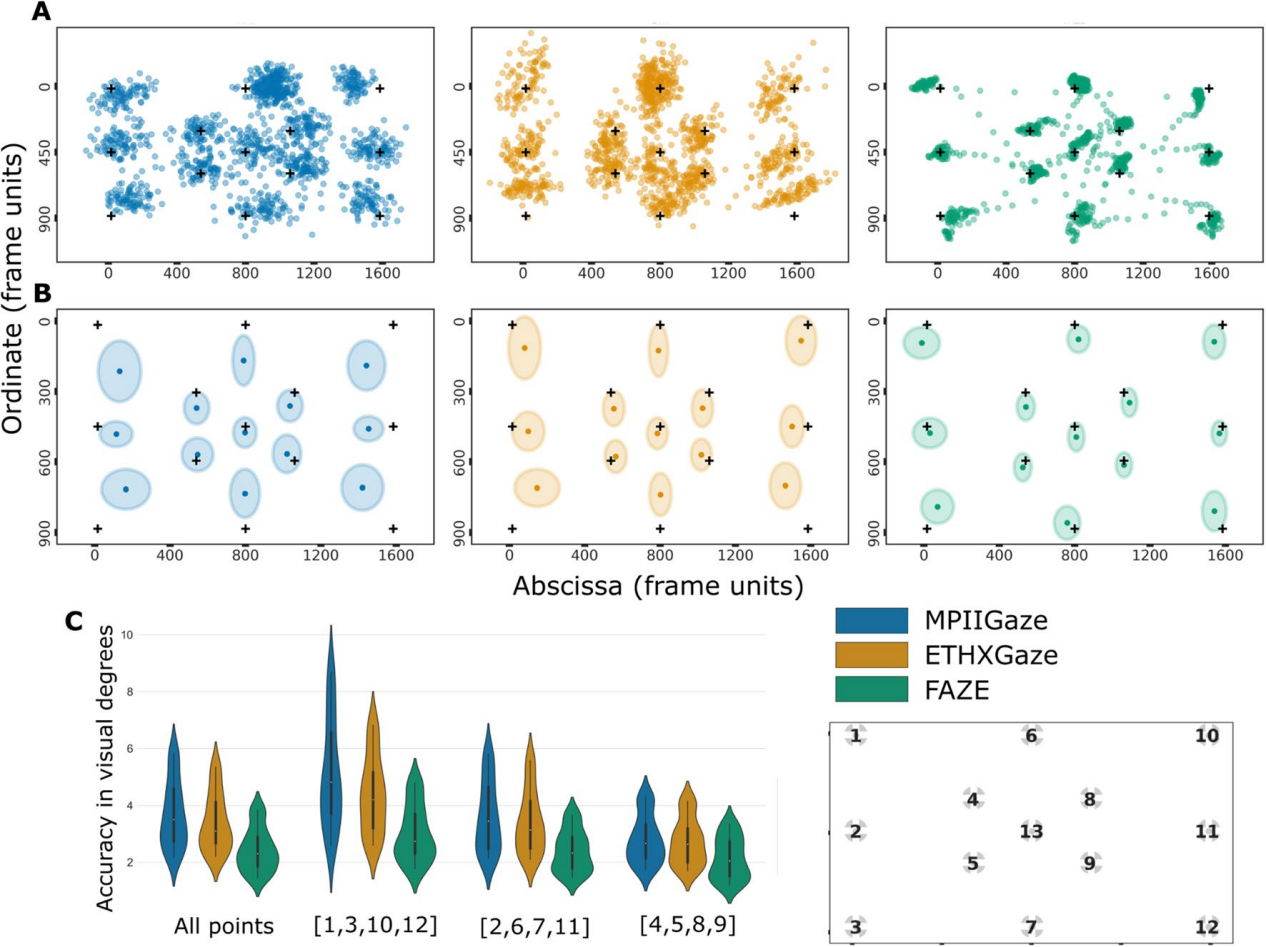
Comparison of gaze predictions from the three deep learning models on the *fixation* *task*. **A** Single trial raw gaze predictions from each model. **B** Aggregated fixation locations over all 65 participants. Crosses represent the displayed fixation target locations; dots represent mean predicted fixations. The ellipse axes represent standard deviation in *x* and *y* directions, respectively. **C** Mean fixation accuracy accumulated over all locations, compared to accuracy over locations presented in the corners of screen [1,3,10,12], center of screen edges [2,6,7,11], and near the center of screen [4,5,8,9]; see coding of locations by numbers at the right part of Fig. 2C

We calculate accuracy and precision measures for the *fixation* *task*. Accuracy refers to the offset between displayed target and estimated gaze prediction, while precision captures consistency in recorded data by taking into account the dispersion within predicted gaze points. Fixations were estimated as medians of raw gaze predictions (see examples in Fig. 2, top row) over the 2500-ms target display duration. For precision, we calculated the root-mean-square (RMS) and standard deviation (STD) between gaze samples. RMS measures inter-sample distances and is calculated as the square root of the mean of squared distances between consecutive samples. STD provides a more intuitive measure of the precision as the spread of gaze point predictions around the mean. It is calculated as the square root of the mean of squared distances of each sample from the mean fixation location (x̄, ȳ). Good precision reflects less variance (noise) and more reliability in predicted gaze points. In the case of both accuracy and precision metrics, a lower value is better. The final accuracy calculated in such tasks is significantly dependent on the velocity or dispersion thresholds of the applied identification algorithm. Final measures were aggregated by calculating winsorized means, first over all 13 locations, then over the ten trials, and finally over all 65 participants.

$$\begin{array}{ccc}\text{RMS}:\sqrt{\frac1n\sum\nolimits_{i=1}^n\left(\left(x_i-x_{i-1}\right)^2+\left(y_i-y_{i-1}\right)^2\right)}&&\;\text{STD}:\sqrt{\frac1n\sum\nolimits_{i=1}^n\left(\left(x_i-\bar x\right)^2+\left(y_i- \bar y\right)^2\right)}\end{array}$$

#### Zone classification

This task provides a less strict measure of gaze prediction accuracy and resembles tasks that define "regions-of-interest" for gaze locations. The performance for this task was evaluated by the accuracy of correctly classifying gaze predictions to the viewed zone. Raw gaze predictions over a presentation trial were aggregated for each of the 16 presented locations (zones) as the median of gaze points for the 1500 ms duration the pattern was presented in that zone. For each participant, classification accuracy over the ten trials was evaluated by classifying the calculated medians to one of the 16 zones based on their position on the grid. Accuracy is higher when predicted zones (calculated medians) are classified to the corresponding presented zones highlighted to the participant. Final accuracy was calculated as the winsorized mean accuracy over all 65 participants. A confusion matrix, aggregated over all 65 participants, demonstrates the winsorized mean accuracy of classifying the predicted zone to the presented zone. We also report average precision for the classification analysis, that is, the ratio of times a zone was correctly identified out of all instances that zone was predicted.

We calculated classification measures using raw gaze predictions and strictly defined zone boundaries; however, it is important to note that classification accuracy is highly dependent on classification criteria. For example, Akinyelu & Blignaut, 2020 proposed a “Loosely Correct Estimation Rate” measure, which allows lenient boundaries for zones, accounting for misclassified gaze points near the boundary and therefore improves final classification accuracy.

#### Free viewing

The *free-viewing* *task* evaluates model predictions in unrestricted gaze movement scenarios where participants were free to view anywhere on the screen. We extracted fixations from the time series of gaze recordings for each presented image. Models were compared on the mean number of fixations recorded in a trial and the gaze entropy of recorded fixations. The calculated fixations were used to compute heatmaps and scanpaths that provide a quantitative measure of eye movement complexity. The calculation of heatmaps and scanpaths is largely dependent on the algorithm used for event-classification in such tasks, which in turn is dependent on the spatiotemporal resolution of the eye tracking data. Since acceleration/velocity thresholds used in standard fixation detection algorithms are not reliable with low sample rates, we applied a modified density-based clustering algorithm (Cakmak et al., 2021), leveraging both spatial and temporal domain features to detect fixations. The spatial density threshold was defined as half of the calculated RMS precision values in the *fixation* *task* for each model (MPIIGaze: 1.13°, ETHXGaze: 0.89°, FAZE: 0.26°), and the temporal threshold was set to six samples (~ 200 ms) (Manor & Gordon, 2003). Fixation clusters had a minimum size of two samples and were defined by their centroid (*x*, *y*) location. The first fixation cluster was discarded for each trial to reduce the effect of initial center fixation. Detected fixation clusters were used to calculate scanpaths and gaze heatmaps. Continuous gaze heatmaps from the eye tracking data were obtained by convolving a Gaussian filter across participant fixations, with sigma equal to the approximate size of fovea i.e., one degree of visual angle to match the filter size used in Judd et al. (2009).

#### Smooth pursuit

The *smooth pursuit* *task* records dynamic eye movements generated when the eyes fixate on a moving target. Performance on this task was evaluated using the mean deviation of predicted gaze movement angle from the target movement angle. Raw gaze predictions during the smooth pursuit target movement duration were extracted and smoothed using a Savitsky Golay filter (order = 1, window length = n/2, n = length of gaze sequence). We estimated the angle of eye movement by fitting a linear regression model on the smoothed predictions. For each trial, deviation from the target movement was calculated as the absolute difference between the estimated gaze direction and target movement angle. Final scores were aggregated by calculating winsorized means, first over all 12 target movement angles, then over all ten trials, and finally over all 65 participants.

We also applied custom event detection algorithms to calculate the smooth pursuit movement onset latency and duration. Event detection for 30-Hz gaze-tracking data is a complicated task, as the traditional velocity and density-based algorithms used with laboratory-based eye trackers are not suitable. Here, we applied time series analyses to identify onset and offset of the smooth pursuit in the critical interval between self-paced initiation of the trial by mouse click and the stop of the target movement. Raw gaze data were smoothed using a Savitsky Golay filter (order = 1, window length = n/3, n = length of gaze sequence), followed by offline changepoint detection on the first derivative time series of the smoothed gaze points, using the ruptures library (Truong et al., 2018). The changepoint detection algorithm finds an optimal segmentation of the time series using two changepoints by computing costs for all possible subsequences identified with a dynamic programming search method (minimum distance between changepoints was set to three). The two changepoints marked the onset and offset of smooth pursuit movement in each trial. These measures allowed us to also perform additional temporal analysis and compare performance of our method with laboratory-based eye tracking methods, which was not feasible initially (see Analysis plan in Saxena et al., 2021).

#### Blink detection

For this task, a series of “Eye Aspect Ratio” values and blink probabilities were predicted using the two methods EAR and RT-BENE, respectively. The two time series were used to calculate the number of detected blinks per trial. Given the task requirements, we expected the number of blinks per trial to be seven, and temporally close to the auditory cue.

Similar to smooth pursuit, we applied offline change-point detection with the ruptures library (Truong et al., 2018) to the two resulting time series (from each model) for detecting potential blink onsets or offsets. To optimize computation with the longer blink time series we used the linearly penalized segmentation (Killick et al., 2012). Then, we eliminated false positives by making sure there was in fact a peak detected after a change point. If no peak was identified within the neighboring six frames (~ 200 ms) of a change-point, that point was discarded as a false identification. Filtered change-points were then clustered using agglomerative clustering (distance threshold = 25 frames) to classify identified change-points as onset and offset of each blink. In addition to the number of blinks detected per trial, we calculate blink latencies as the time offset between beep onset and detected blink onset, as well as blink durations as the time difference between blink offset and onset.

## Results

### Fixation

Raw gaze predictions from all three models for a single trial are presented in Fig. 2A. We compared fixation accuracy from the three models using a one-factorial, repeated measures ANOVA, *F*(2, 128) = 13.10, *p* < 0.01, *η*^2^ = 0.09. Post hoc *t* tests found significant differences between MPIIGaze and FAZE *t*(64) = 9.79, *p* < 0.01, and ETHXGaze and FAZE, *t*(64) = 3.69, *p* < 0.01, but not between ETHXGaze and MPIIGaze, *t*(64) = 0.32, *p* = 0.75. Overall accuracy was 3.70° (IQR: 2.74–4.59°) for MPIIGaze, 3.40° (IQR: 2.67–4.13°) for ETHXGaze, and 2.44° (IQR: 1.86–2.90°) for FAZE. Figure 2B plots the mean fixation accuracy at each target location for the three models. Accuracy was much better at the center of the screen, getting worse towards the screen edges. We followed up on this effect of eccentricity with a post hoc ANOVA, including models as one factor and three eccentricity ranges (see Fig. 2C) as a second factor. The main effect of model was significant, *F*(2, 128) = 55.22, *p* < 0.01, *η*^2^ = 0.15, as was the main effect of eccentricity, *F*(2, 128) = 266.71, *p* < 0.01, *η*^2^ = 0.21. The interaction was also significant, *F*(4, 256) = 31.35, *p* < 0.01, *η*^2^ = 0.02. The differences in accuracy between the three eccentricities were more pronounced for MPIIGaze and ETHGaze models, as compared to FAZE; however, the order remained the same, i.e. accuracy at locations [1,3,10,12] > [2,6,7,11] > [4,5,8,9], for all three models, with statistically significant differences for all comparisons, all *t*’s > 5.44, all *p*’s < 0.01. One factorial ANOVA comparisons on the RMS and STD values of the three models showed a significant effect of model (RMS: *F*(2, 128) = 409.33, *p* < 0.01, *η*^2^ = 0.63, and STD: *F*(2, 128) = 183.89, *p* < 0.01, *η*^2^ = 0.30). Post hoc *t* tests revealed significant differences between all three models, all *t*’s > = 8.36, all *p*’s < 0.01 (detailed report of all post hoc statistics available in the code repository associated with this paper). Winsorized mean RMS was 2.33° (IQR: 1.88–2.75) for MPII, 1.80° (IQR: 1.28–2.29°) for ETH, and 0.47° (IQR: 0.29–0.64) for FAZE. Winsorized mean STD was 2.96° (IQR: 2.32–3.57°) for MPII, 2.39° (IQR: 1.70–2.98) for ETH and 1.63°(IQR: 1.06–2.07°) for FAZE.

### Zone classification

**Fig. 3:**
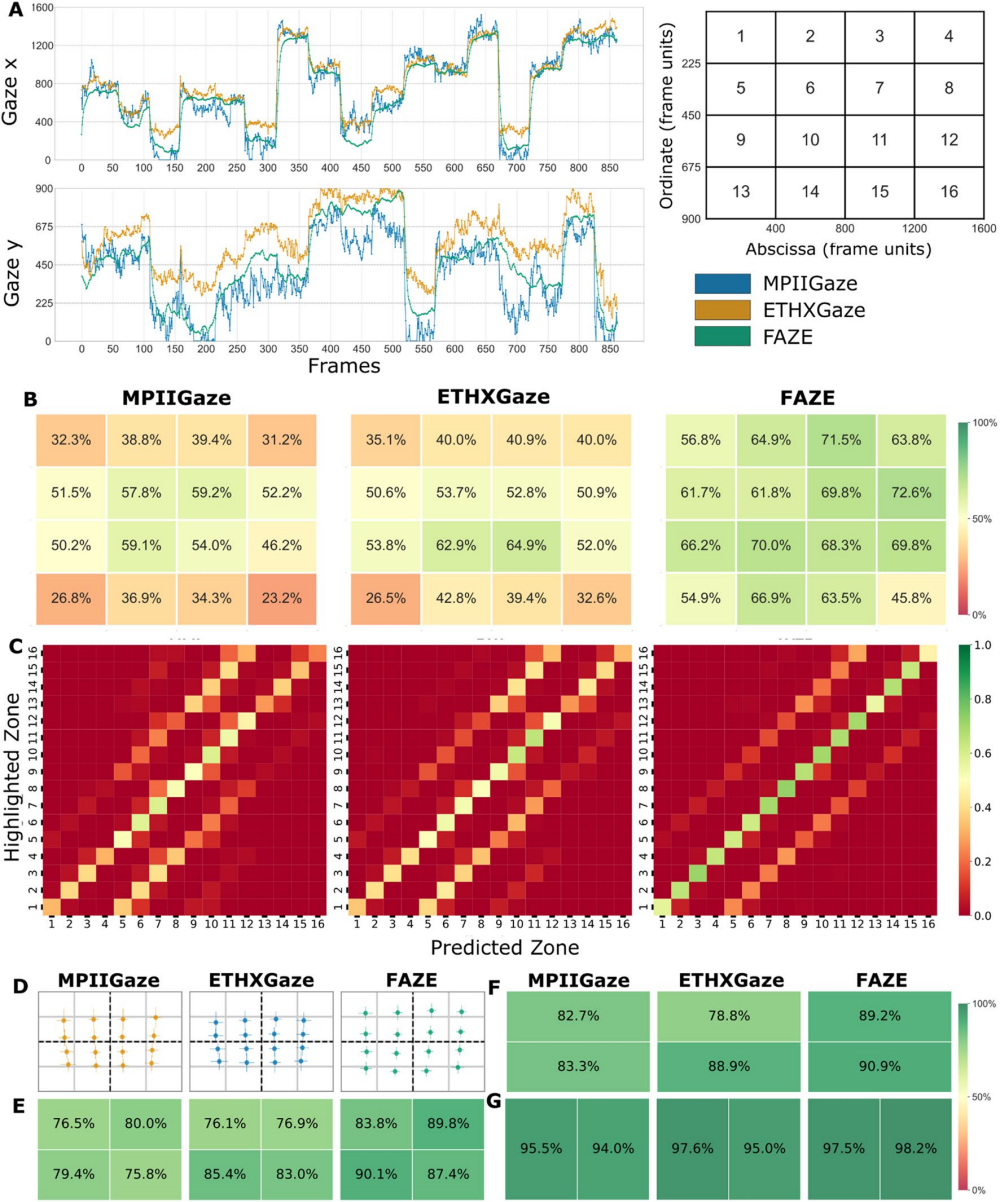
Comparison of gaze predictions from the three models on *zone classification* accuracy. **A** Single trial raw gaze predictions, represented as a time series of *x* (left panel) and *y* (right panel) coordinates. All the models capture similar eye movement patterns, but with differing levels of noise in their predictions. **B** Gaze classification accuracy for each zone independently. Classification accuracy is highest in the center of the screen as observed in the *fixation* *task*. **C** Classification matrix for each model (MPIIGaze, ETHXGaze and FAZE). Please find the coding of grid locations as numbers in the upper right of this Fig. 3. The diagonal represents the correct classifications of predicted zones to the corresponding highlighted zone. Misclassifications are predominantly made in neighboring zones, highlighted by lines parallel to the diagonal. **D** Winsorized mean gaze prediction over all participants, for each zone presentation. **E** Classification accuracies for grid size of 2 × 2. **F** Classification accuracies for grid size of 1 × 2. **G** Classification accuracies for grid size of 2 × 1

Figure 3A shows raw single trial gaze time series (*x* and *y* coordinates) from all three gaze prediction models. Zone-wise classification accuracy (can vary between 0 to 1 with 1 being 100% accurate), aggregated over all participants, is presented in Fig. 3B. All models capture the gaze movements in a similar pattern, however, with varying accuracy and precision, as was also seen in the *fixation* *task*. A one-factorial ANOVA identified a significant effect of model on classification accuracy, *F*(2, 128) = 34.44, *p <* 0.01, *η*^2^ = 0.16, with FAZE being significantly better than the other two models (MPII and FAZE: *t*(64) = 7.63, *p <* 0.01, and ETH and FAZE: *t*(64) = 7.38, *p <* 0.01). FAZE predictions provided the best classification accuracy (accuracy = 0.65 and precision = 0.70), followed by ETHXGaze (accuracy = 0.46 and precision = 0.53) and then by MPIIGaze (accuracy = 0.43 and precision = 0.48). Further analyzing the classification matrix (Fig. 3C) we find that misclassifications for a zone were made mostly for its neighboring zones, particularly the vertical neighbors marked by parallel diagonals in the matrix. 

 In contrast to aggregating computed classification measures from all participants, we also performed stimulus-level analysis by aggregating gaze predictions over all trials and participants for each zone (see Fig. 3D). The classification accuracy in this case was 0.53 for MPIIGaze (average precision = 0.69), 0.54 for ETHXGaze (average precision = 0.67), and 0.76 for FAZE (average precision = 0.81). We again found classification errors to be highly concentrated in the neighboring zones and proceeded to quantify the classification accuracy improvement with a larger zone. We, therefore, calculated classification accuracy on different levels of grid division, varied by changing the size of the grid to 2 × 2 (Fig. 3E), 1 × 2 (Fig. 3F), and 2 × 1 (Fig. 3G), where W × H grid size represents divisions across the width (W) and divisions across the height (H) of the screen. Larger zones increased classification accuracy and precision for all three models (see Fig. 3E–G and Table 1). Consistent with the higher number of misclassifications made for vertical neighbors in a 4 × 4 grid, we find that for binary screen splits, the 2 × 1 grid yielded higher accuracy than 1 × 2 (see Fig. 3F, G).

**Table 1:** Winsorized mean model accuracy and precision for different zone size divisions

	Accuracy			Precision		
	2 × 2	2 × 1	1 × 2	2 × 2	2 × 1	1 × 2
MPIIGAZE	0.78	0.94	0.82	0.80	0.95	0.84
ETHXGAZE	0.80	0.96	0.83	0.83	0.96	0.86
FAZE	0.88	0.98	0.89	0.90	0.98	0.91

Zone divisions are represented according to WxH, where W represents the number of divisions across the width of the screen and H represents the divisions across the height of the presentation screen

### Free viewing

**Fig. 4:**
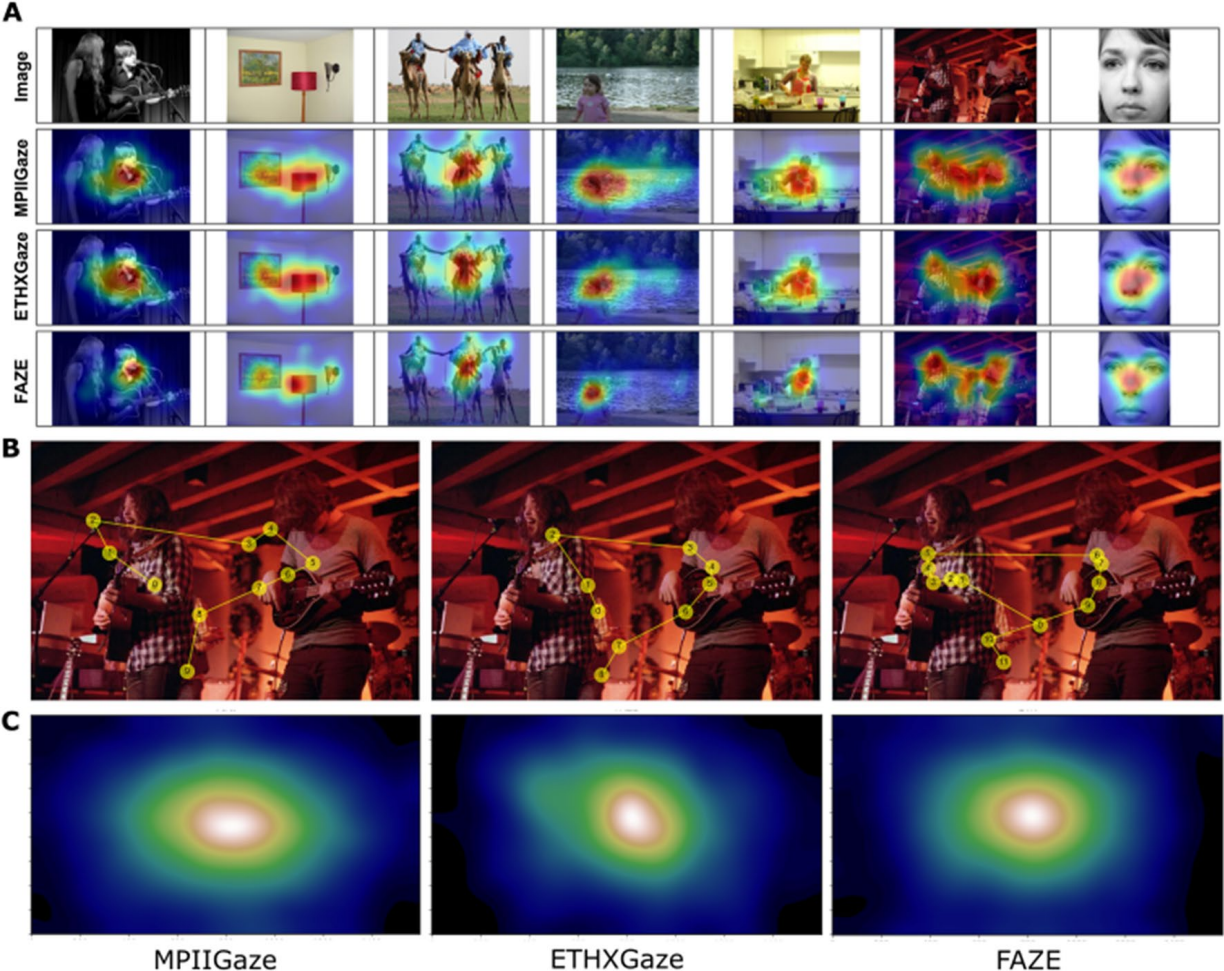
Analysis of gaze prediction of the three models on the *free-viewing* *task*. **A** Gaze heatmaps (rows 2–4) for a sample of images (row 1) from the Judd dataset (Judd et al., 2009). Heatmaps calculated from FAZE predictions represent discrete clusters better than ETHXGaze and MPIIGaze. **B** Example scan paths calculated for a single trial from a single participant, based on the sequence of fixation locations. **C** Average gaze heatmaps calculated from fixations of all participants over all images

In Fig. 4A, gaze heatmaps from the fixations identified by the three models are presented for a subset of *free-viewing* *task* images. The calculated fixation locations for a single trial could be used to plot scan paths (Fig. 4B), providing a visual representation of the gaze prediction performance of the three models. One factorial ANOVAs identified a significant effect of model on the mean number of fixations recorded in a trial, *F*(2, 128) = 20.86, *p <* 0.01, *η*^2^ = 0.09 and the mean gaze entropy, *F*(2, 128) = 48.46, *p <* 0.01, *η*^2^ = 0.17. MPIIGaze recorded a significantly higher number of fixations (M = 14, IQR: 12–18) than ETHXGaze (M = 13, IQR: 10–15) and FAZE (M = 12, IQR: 11–13). Meanwhile the mean entropy for MPIIGaze was M = 0.40 (IQR: 0.38–0.44), for ETHXGaze M = 0.38 (IQR: 0.35–0.41), and for FAZE M = 0.35 (IQR: 0.33–0.37), with significant differences between each pair, all *t*’s > = 4.70, all *p*’s < 0.01. Averaging gaze heatmaps from all images outlined the classic central fixation bias (Tatler, 2007) as shown in Fig. 4C.

### Smooth pursuit

**Fig. 5:**
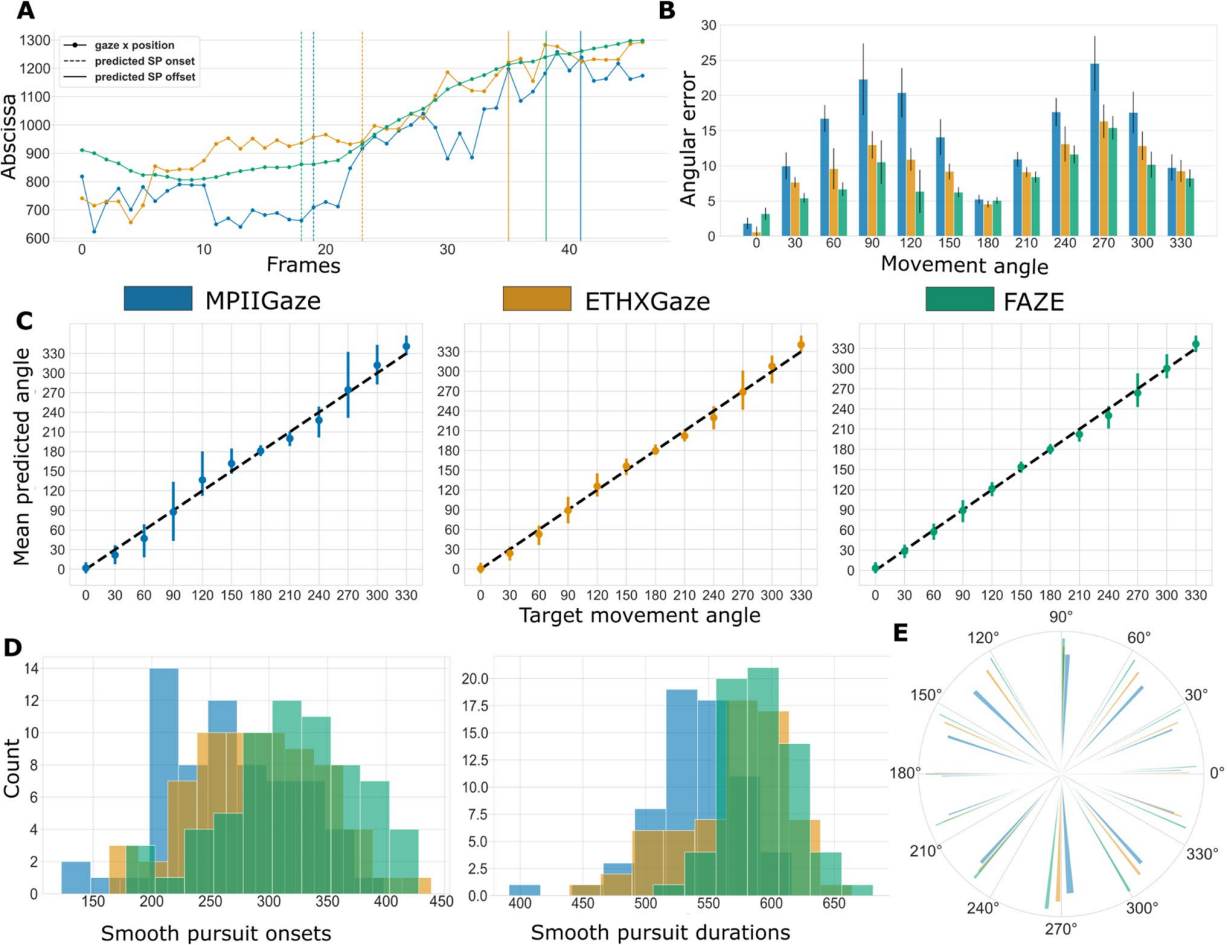
Analysis of smooth pursuit movements from the gaze prediction of all three models. **A** X coordinates of a single trial gaze prediction time series from all three models. The target movement for this trial was in the horizontal direction (0°). Dashed lines (--) represent predicted onset of smooth pursuit movement; continuous vertical lines represent predicted offset. **B** Error in predicted gaze movement, calculated as the difference between predicted gaze angle and the target movement angle for each trial. The difference was highest when the target moved in vertical directions (90° and 270°) and lowest when the target moved horizontally (0° and 180°). **C** Winsorized mean predicted angle over all trials for each target movement. **D** Histograms of predicted onsets and durations of smooth pursuits for each of the models. **E** Mean angle of gaze movement over all trials and participants. The width of the bars represents the standard deviation for each angle and the colors/length represent the different models

Figure 5A shows the x dimension of gaze predictions for a single trial where the target moved along the horizontal axis (movement angle = 0°), along with estimated movement onsets and offsets (vertical lines). We compared angular deviations from the three models using a one-factorial, repeated measures ANOVA, *F*(2, 128) = 9.36, *p <* 0.01, *η*^2^ = 0.06. Post hoc *t* tests showed significant differences between MPIIGaze and ETHXGaze, *t*(64) = 2.35, *p* = 0.02, and between MPIIGaze and FAZE, *t*(64) = 4.16, *p <* 0.01. The aggregated deviation for the three models were, MPIIGaze: 13.39° (IQR: 17.03–7.37°), ETHXGaze: 9.55° (IQR: 11.88–6.99°) and FAZE: 8.09° (IQR: 9.97–5.34°). Angle-wise deviations can be seen in Fig. 5B below. The highest error in predicted gaze directions occurred when the target moved vertically (90° or 270°) on the screen. Predicted angles over all trials and participants are presented in Fig. 5C and E. There was a significant effect of model on calculated onset latencies, *F*(2, 128) = 47.42, *p* < 0.01, *η*^2^ = 0.14, and smooth pursuit durations, *F*(2, 128) = 52.12, *p* < 0.01,* η*^2^ = 0.24. Mean onset latencies for smooth pursuit movement (Fig. 5D) were: M = 267.16 ms (IQR: 215.04–307.08 ms) for MPIIGaze, M = 291.42 ms (IQR: 251.87–330.95 ms) for ETHXGaze, and M = 323.58 ms (IQR: 292.03–368.61 ms) for FAZE predictions, with significant differences between each pair, all *t*’s > 4.56, all *p*’s < 0.01. The mean smooth pursuit durations (Fig. 5D) calculated from the onset and offsets were: 539.86 ms (IQR: 524.12–563.01 ms) for MPIIGaze, 569.86 (IQR: 544.01–599.19 ms) for ETHXGaze and 587.91 ms (IQR: 568.48–607.25) for FAZE predictions, with significant differences between each pair, all *t*’s > = 3.76, all *p*’s < 0.01.

### Blink detection

**Fig. 6:**
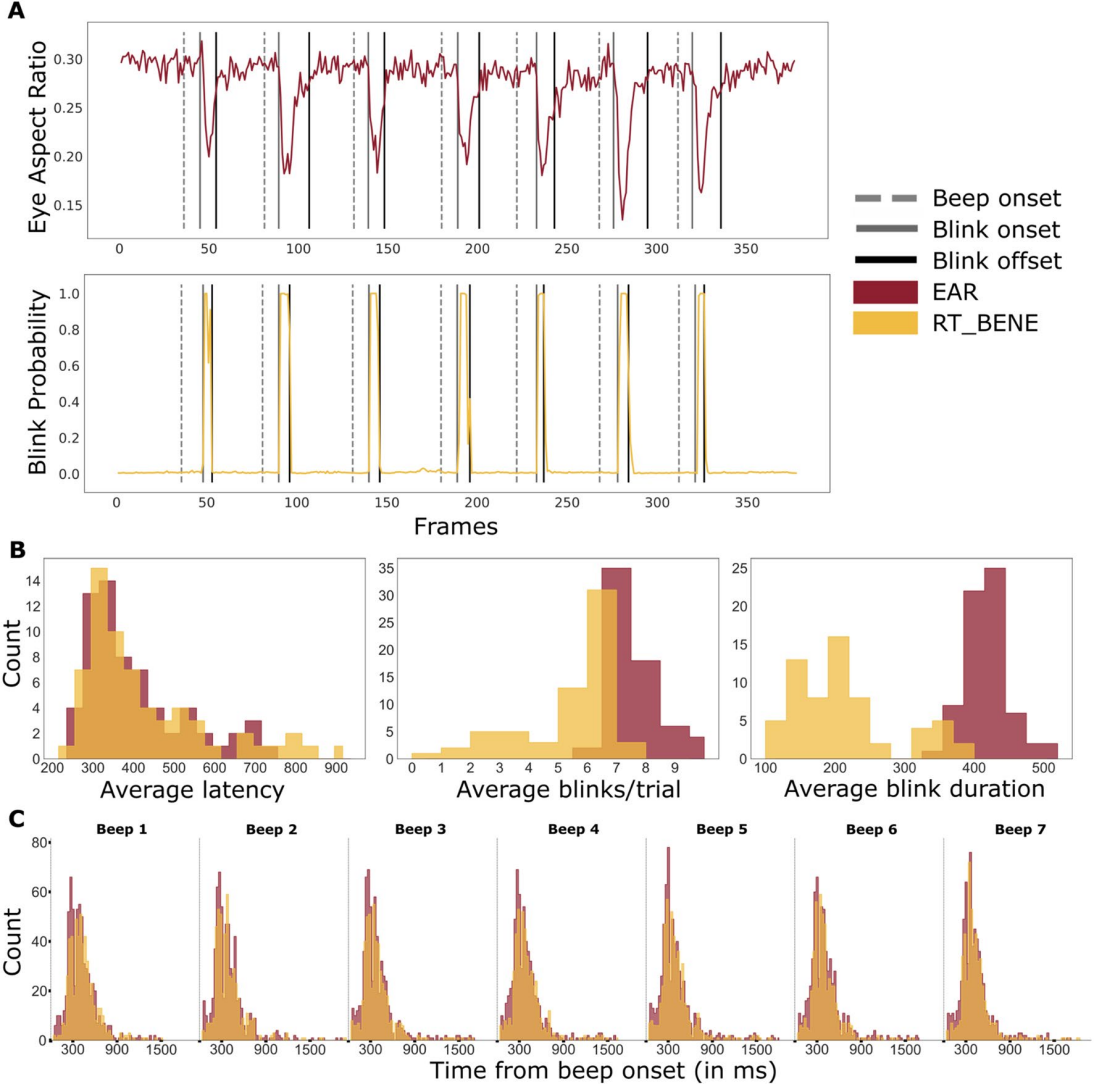
Comparison of blink predictions from the two blink models. **A** Single trial raw blink predictions from the RT_BENE and EAR methods. Black lines represent predicted blink offsets and grey lines represent predicted blink onsets. **B** Histograms of average blink onset latency, number of blinks per trial, and blink duration, over all participants, calculated for both models. **C** Blink onset latency distributions for each of the cued blinks separately. Dashed lines represent auditory beep (cue) onset

Figure 6A shows the predicted time series from the two blink detection methods for a randomly selected trial. The average number of blinks detected per trial by the two methods was found to be significantly different using a one-factorial, repeated measures ANOVA, *F*(1, 61) = 54.72, *p <* 0.01, *η*^2^ = 0.36. The number of blinks per trial was 6.9 (IQR: 6.3–7.2) for EAR and 5.3 (IQR: 4.9–6) for RT_BENE. The winsorized mean onset latency of blinks was 381 ms (IQR: 293–427 ms) for EAR and 419 ms (IQR: 318–502 ms) for RT_BENE (see Fig. 6B, first column). The winsorized mean duration of blinks was 403 ms (IQR: 383–424 ms) for EAR and 212 ms (IQR: 157–243 ms) for RT_BENE (see Fig. 6B, third column). Figure 6C shows the detected blink onsets after the auditory beep onset time for all seven beeps.

### Comparison of online webcam results to in-laboratory eye tracking

The *fixation, smooth pursuit*, and *blink detection* *tasks* in our study were implemented as similarly as possible to the corresponding tasks in Ehinger et al., 2019. Their study simultaneously evaluated two eye trackers with a higher sampling rate (SR Research’s EyeLink 1000: sampling rate at 500 Hz; Pupil Labs’ Pupil Core glasses: sampling rate at 120 Hz), in a laboratory-based setup. We compare their reported measures on these tasks with the best performing model/strategy of our webcam-based methods in Table 2. It is not astonishing that eye trackers outperform the webcam estimations, as the first have a much higher spatial and temporal resolution. Moreover, a fair comparison would require simultaneous recording from all methods. We provide these comparisons, nevertheless, to document differences between eye trackers and webcam deep learning methods, as well as the unexpected high consistency regarding timing (smooth pursuit, blink detection).

**Table 2:** Comparison of webcam results (means) from this study and published results based on high-speed laboratory-based eye trackers (PL: Pupil Labs’ Pupil Core glasses and EL: SR Research’s EyeLink 1000)

Task measure	Webcam results		Laboratory-based results		Comparisons
**Fixation**					
Accuracy	MPIIGaze	3.70°	PL	0.82°	*t = 10.61, p < 0.01*
	ETHXGaze	3.40°	EL	0.57°	*t = 12.12, p < 0.01*
	FAZE*	2.44°			
Precision (RMS)	MPIIGaze	2.33°	PL	0.119°	*t = 13.47, p < 0.01*
	ETHXGaze	1.80°	EL	0.023°	*t = 17.02, p < 0.01*
	FAZE*	0.47°			
Precision (STD)	MPIIGaze	2.96°		0.311°	*t = 14.97, p < 0.01*
	ETHXGaze	2.39°	EL	0.193°	*t = 16.29, p < 0.01*
	FAZE*	1.63°			
**Smooth pursuit**					
Onset latency	MPIIGaze*	0.267 s	PL	0.245 s	*t* = 2.84, *p* < 0.01
	ETHXGaze	0.291 s	EL	0.241 s	*t = 3.40, p < 0.01*
	FAZE	0.323 s			
**Blink detection**					
Number of blinks	EAR*	6.9	PL	5.3	*t = 15.7, p < 0.01*
	RT_BENE	5.3	EL	7.1	*t* = 0.98, *p* = 0.3
Blink duration	EAR	0.403 s	PL	0.214 s	*t = 0.15, p = 0.88*
	RT_BENE*	0.212 s	EL	0.190 s	*t* = 2.40, *p* = 0.02

One-sample *t* tests compare the webcam results of the closest model (marked by *) with the respective laboratory-based eye-tracker results

For the *free-viewing task*, we computed another measure of accuracy by comparing the heat maps from our gaze predictions to the fixation heat maps from Judd et al. (2009). For relating the spatial locations and features of the heat maps, we calculated three indices: Area under ROC curve (AUC), Pearson’s correlation coefficient (CC), and similarity (SIM) scores and report averages for the 16 images. Mean AUC scores were M = 0.50 (IQR: 0.47–0.52) for MPIIGaze, M = 0.53 (IQR: 0.51–0.56) for ETHXGaze, and M = 0.61 (IQR: 0.58–0.63) for FAZE. Mean CC scores were = 0.46 (IQR: 0.43–0.49) for MPIIGaze, = 0.47 (IQR: 0.42–0.52) for ETHXGaze, and M = 0.58 (IQR: 0.54–0.63) for FAZE. Mean SIM scores were M = 0.37 (IQR: 0.35–0.38) for MPIIGaze, M = 0.38 (IQR: 0.36–0.41) for ETHXGaze, and M = 0.43 (IQR: 0.41–0.46) for FAZE. For all three indices, the models differed significantly and FAZE was better than the other two models, all *t*’s > = 4.62, all *p*’s < 0.01. As before, please note that the fixation heatmaps taken as ground truth were recorded with much higher temporal resolution, under highly controlled environments.

### Model comparisons across tasks

**Fig. 7:**
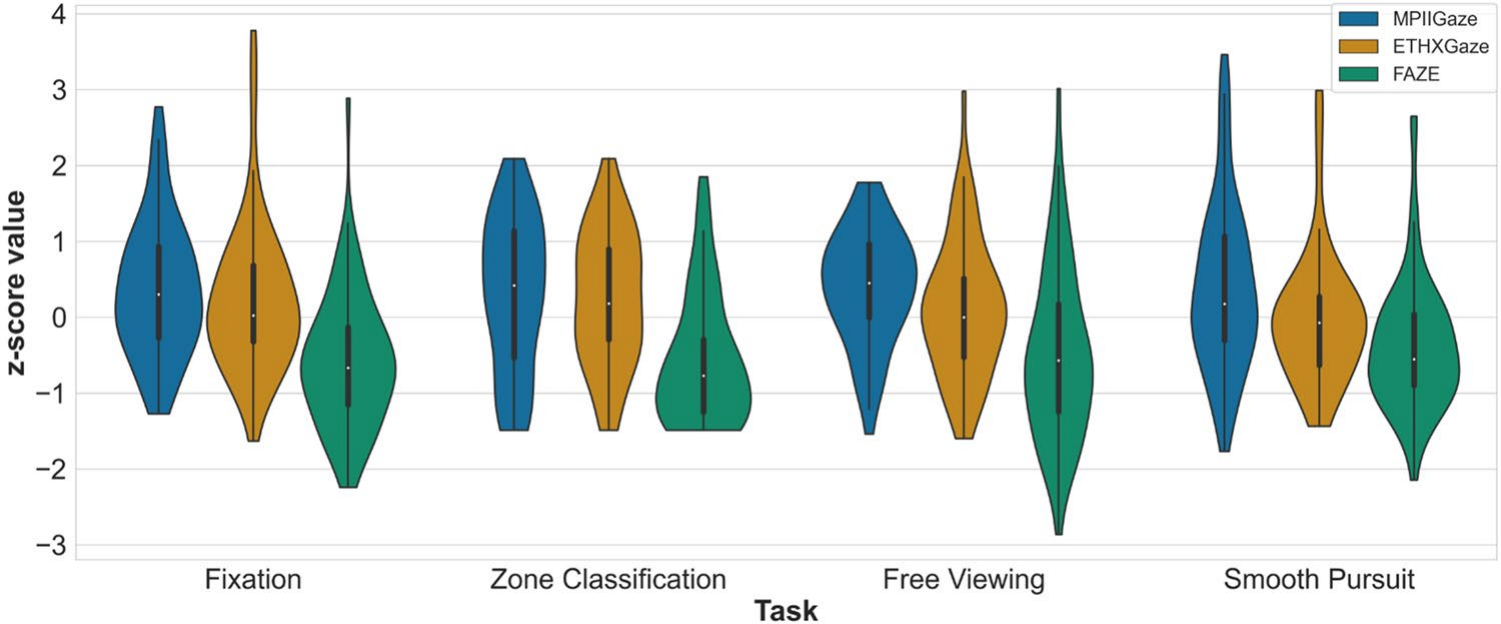
Model-wise distribution of transformed scores for each task

We selected single measures from each task to represent task performance. We used accuracy from the *fixation task*, classification accuracy from the *zone classification task*, average AUC score from *free viewing*, and angular deviation from the *smooth pursuit* *task*, to compare the performance of all three gaze prediction models between all four tasks, with a 3 × 4 ANOVA. Note that all selected measures evaluate performance of the three models on the task parameters, with the exception of AUC score for the *free-viewing* *task*, which compares each model’s performance to the computed heatmaps from the original study (Judd et al., 2009). Nonetheless, all three models were compared to the same ground truth, providing a measure of accuracy of the three deep learning models relative to each other on this task. There was a significant main effect of model, *F*(2, 128) = 57.12, *p <* 0.01, *η*_p_^2^ = 0.04. Task did not have a significant effect on performance, and there was also no interaction between the task and model factors. Overall, FAZE outperformed ETHXGaze, *t*(64) = 7.75, *p* < 0.01 and MPIIGaze, *t*(64) = 10.88, *p* < 0.01, and ETHXGaze outperformed MPIIGaze, *t*(64) = 2.47, *p* = 0.05 (see Fig. 7 for accuracy distribution over tasks).

## Discussion

We are the first to systematically assess the application of appearance-based, deep learning, gaze and blink estimation models in online experiments. Combined with our calibration strategies (see Saxena et al., 2022, and Supplementary Information), the proposed methods achieve a fixation accuracy of 2.4° and precision of 0.47° with the FAZE model (Park et al., 2019), making webcam eye tracking a robust addition to online research. In comparison with web-browser-based eye tracking performance reported in previous studies (Bánki et al., 2022; Papoutsaki et al., 2016; Semmelmann & Weigelt, 2018; Yang & Krajbich, 2021) with accuracy ranging between 3–4°, this accuracy is substantially better and further reduces the gap between online and laboratory-based eye tracking. We provide our experiment template, recorded data, and analysis code, including novel event-detection algorithms for low resolution eye tracking data, to aid future research and applications.

Our task battery utilized common eye tracking tasks to characterize performance of three gaze-estimation models: MPIIGaze (Zhang et al., 2015), ETHXGaze (Zhang et al., 2020) and FAZE (Park et al., 2019), along with two blink detection methods: EAR (Soukupová & Cech, 2016) and RT-BENE (Cortacero et al., 2019). The most common measures of eye-tracker performance are accuracy and precision, which we calculated using the *fixation task*. Consistent with common eye-tracker reports, accuracy was found to be better near the center of the screen, reducing as gaze moved toward the screen edges (Fig. 2C). The degree of this eccentricity effect was, however, dependent on the model, indicating that choice of model architecture can resolve this inconsistency. Interestingly, the best and worst prediction accuracy for each model was for different subjects (see Supplementary Fig. 2), highlighting each model’s differing reliance on appearance features, such as lighting, camera angle, reflections, skin/eye color etc. Model architecture and pipeline also has a significant effect on the reliability of reproducing gaze predictions during a fixation, represented by the precision measure.

The *zone classification task* replicates another common experimental paradigm (preferential looking) in psychological studies that requires participants to shift gaze to specific regions-of-interest on the screen, instead of small fixation targets. The best performing model, FAZE (Park et al., 2019), achieved 65% classification accuracy for a 4 × 4 grid. Studies on visual attention often apply preferential looking paradigms to regions-of-interest, but typically with lower numbers of regions and/or spatially larger areas, which are placed along the vertical or horizontal axis of the screen (e.g., Liu et al., 2011; Schofield et al., 2012). Therefore, we evaluated classification accuracy also for the underlying grids 2 × 1, 1 × 2, 2 × 2. Indeed, accuracy was higher when the size of the areas was increased (e.g., FAZE = 98% for 2 × 1 grid). Importantly, classification error was higher for vertical divisions depicted by the higher accuracy for the 2 × 1 as compared to 1 × 2 grid (Fig. 3F and 3G), misclassified zones in 4 × 4 grid (Fig. 3C), and single trial data (Fig. 3A). Researchers should therefore prefer horizontal left-right over vertical top-down screen-splits for similar paradigms.

Our proposed event-detection algorithms robustly identified fixations for 30-Hz eye tracking recording during the *free-viewing task*. This identification of fixations was important, as including raw gaze points during saccades results in an overestimation of the central fixation bias in the heat maps. Therefore, our heat maps were based on fixations only. We found discernable clusters of salient objects in the generated heatmaps. Salient clusters in generated heatmaps and the shape of scanpaths were noticeably different for each model, highlighting the qualitative differences between their gaze predictions. Despite this dissimilarity, all three models identified a comparable number of fixations and gaze entropy over the presented images, even though they differed in statistical terms. Importantly, FAZE outperformed the other models when comparing the computed heatmaps with the recorded fixation heatmaps of the original study, which were recorded with a high-speed eye tracker with high resolution (Judd et al., 2009). These results demonstrate the utility of the current method for studying visual behavior and saliency with visual stimuli.

In the *smooth pursuit task*, gaze movement angles were accurately predicted with as low as 8.09° error (FAZE). We found higher error for vertical (Fig. 5B), compared to horizontal, smooth pursuit movements, replicating a known asymmetry of smooth pursuit movements (Ke et al., 2013). Similar to the *free-viewing* analysis we developed customized event detection algorithms (see *Smooth pursuit* in Task measures) that robustly detect smooth pursuit onsets and offsets. Even with the low sample frequency, the calculated onset latencies were comparable to high-speed eye trackers (Table 2). Interestingly, we calculated slightly longer onset latencies with FAZE than the other models, which can likely be attributed to the signal delay introduced by the Kalman filtering in the model pipeline.

Beyond gaze estimation, we evaluated two blink detection algorithms that identified up to 6.9 of the 7 (Fig. 6B) instructed blinks per trial. Typically, blink detection algorithms apply anomaly detection on eye tracking time series data to identify blinks. In contrast to this indirect approach, appearance-based methods compute blink probabilities directly from visual features in recorded video frames, providing better accuracy for lower sampling rates. The proposed methods can be applied independently for accurate blink detection, but also in conjunction with gaze estimation to further improve accuracy by filtering out blinks which are typically represented as irregular or null predictions for samples with partially or completely closed eyes. We again introduce robust methods to analyze and detect blinks from eye tracking time series and make them available for interested readers.

Characterizing model performance over the full task battery provides us with important considerations for future applications and development of gaze-tracking methods. Overall, our proposed method is highly reliable. In particular, the broad range of tasks with very different task requirements and eye movement behaviors demonstrate the utility of our method. Webcam-based eye tracking has the potential to make conventional laboratory-based eye tracking superfluous, and is already a suitable alternative for the highlighted experimental paradigms. For a majority of task measures, FAZE (Park et al., 2019) outperforms ETHXGaze (Zhang et al., 2020), which in turn outperforms MPIIGaze (Zhang et al., 2015) predictions. However, this increased performance is at the expense of increased computational cost (12 fps for MPIIGaze, 4 fps for ETHXGaze and 1 fps for FAZE, where fps represents inference speed in frames per second) which should be considered by researchers when selecting a model architecture.

In comparison to laboratory-based eye tracking, the methods proposed in this paper escalate scalability and affordability of eye tracking research but suffer in terms of accuracy and precision. Only for *blink detection* is the performance similar to laboratory-based methods, depending on the model and metric of interest. Tasks and measures requiring spatial accuracy below 2.4°, or temporal resolution over 30 Hz still need to rely on eye tracking with specialized hardware. Moreover, the high inference time of these models restricts browser-based gaze-contingent paradigms and calls for special emphasis towards data privacy, from the experimenter. Recent frameworks, such as ONNX Runtime Web and WebGPU accelerate deep learning inference in web-browsers; however, the temporal resolution of eye tracking predictions might be significantly reduced from 30 Hz. A comparative study (Gudi et al., 2020) reported CNN inference speeds as fast as 15 Hz, without GPU acceleration, when using single eye inputs for gaze detection. However, this speed came at the cost of reduced gaze estimation accuracy. Offline inference, on the other hand, ensures high spatiotemporal resolution and provides the ability to re-analyze raw data, apply offline correction, and infer multiple measures such as gaze, blinks, gesture recognition, and motion tracking (Chen & McDuff, 2018; Savchenko et al., 2022) on the same data. These benefits aid the development, optimization, and evaluation of robust computational methods. For instance, we found that updating the default face detection model used in the eye tracking methods resulted in fewer missing data and improved gaze predictions (see section “Gaze and blink detection models”). Such an optimization can be easily made in offline setups but would require re-collection of data for browser-based real-time methods which do not allow recalculation from raw data. An ideal method should, therefore, allow this flexibility of online and offline inferencing which is feasible with the deep learning approach proposed in our paper.

With respect to remote, online studies, we have developed and validated an approach to webcam-based eye tracking that outperforms previously proposed methods. We are enthusiastic about the potential for this approach to offer novel, cross-cultural insights into human perception, attention, and subjective experience. This approach opens possibilities of large-scale data collection with limited time and cost spending over different device types. For example, previous studies have implemented similar methods on mobile phones (Valliappan et al., 2020), and tablets (Krafka et al., 2016), driven by the advancements of selfie-cameras and application-specific integrated circuits on these devices. Moreover, this approach allows researchers to recruit participants from populations that cannot come into the laboratory (e.g., those with mobility limitations, immunocompromisation, the elderly, or those in other countries). In summary, our results improve the state-of-the-art in webcam-based eye tracking for online experiments, encouraging further development of open-sourced, affordable, and scalable eye tracking methods that improve validity of scientific studies.

## Data and code availability

All analysis code and anonymized, summary data tables to recreate the figures and statistical analyses reported in this paper are opensourced at https://github.com/ShreshthSaxena/Eye_Tracking_Analysis.

Given the sensitivity of video images of participant’s faces, we were very clear about our intended data processing procedures and participants’ rights to privacy. We offered three levels of consent to participants in our study, spanning publication of processed data to publication of camera recordings. The complete data including camera recordings for the 16 participants who selected level 3 are shared publicly in the following data repository: https://osf.io/qh8kx

The study was pre-registered (Saxena et al., 2021) and a detailed account of designing and comparing calibration strategies for webcam eye tracking studies was published in Saxena et al., 2022, using a subset of the current sample. A template of the online experiment used for data collection is available at https://www.labvanced.com/page/editors/experimentView/41124 and can be used to replicate the entire experiment.

### Supplementary Information

ESM 1 (DOCX 510 kb)
