# Supplementary material for: Deep learning models for webcam eye tracking in online experiments
Source: Behav Res Methods. 2023 Aug 22;56(4):3487–503. doi: 10.3758/s13428-023-02190-6 (PMC11133145; doi:10.3758/s13428-023-02190-6)
Supplement: Supplementary file 1 — (DOCX 510 kb) [file 13428_2023_2190_MOESM1_ESM.docx]

# Supplementary Information

We used calibration data pooled over the two types of calibration tasks (E and SP) and all calibration time points. This approach was identified as the best calibration strategy in our previous study (Saxena et al. 2022), which compared multiple calibration strategies by varying the sample-size, time of calibration during the experiment (beginning, middle and end), and type of calibration task (E or SP). Since Saxena et al. (2022) only involved a subset of the final dataset, here we re-run all analyses reported in Saxena et al. (2022), with the full, final data set. Supplementary Fig.1 shows our final results, which are described in the subsections below.

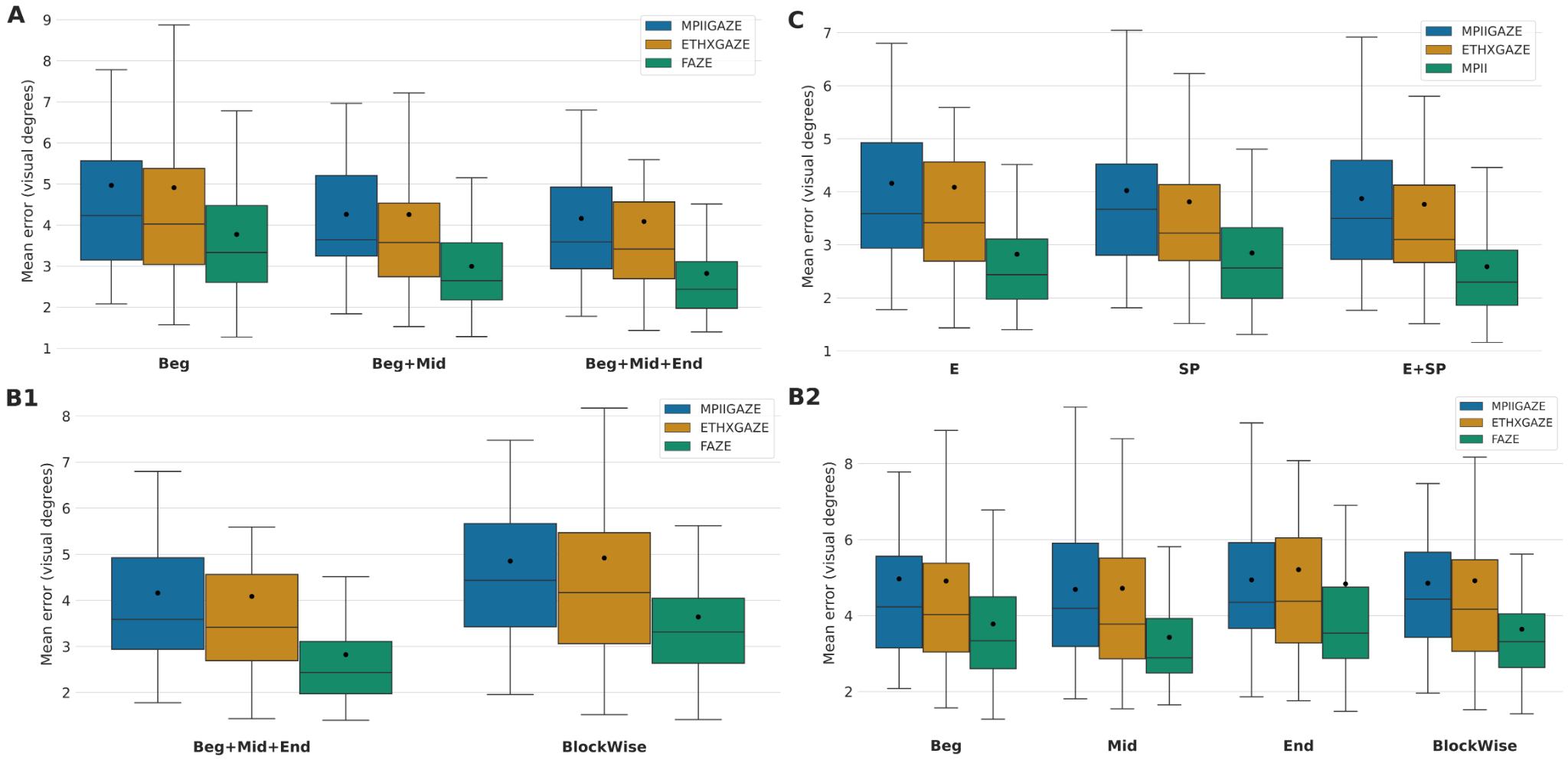


**Supplementary Fig. 1.** Fixation accuracies in different calibration strategies. **A** Comparison of fixation accuracies across different calibration sample sizes. **B1** Comparison of fixation accuracy with best forming calibration sample size (Beg+Mid+End), **B2** Fixation accuracies using single calibration blocks. **C** Fixation accuracies for the two calibration task types (E and SP) and a combined E+SP approach.

## Effect of calibration sample size

A two-factor ANOVA with the factors model type (MPIIGaze, FAZE, ETHXGaze) and sample size (Beg+Mid+End; Beg+Mid; Beg) showed significant main effects of sample size, *F*(2, 128) = 34.169, *p <*  .001, η^2^ = 0.039 and model type, *F*(2, 128) = 9.002, *p <* .001, η^2^ = 0.096. The factors did not interact, *F*(4, 256) = 0.139, *p* = .967, η^2^<0.001. To explore the main effect of sample size, we calculated *t*-tests. Increasing the sample size by adding the Mid calibration block to the Beg, improved accuracy, (Beg and Beg+Mid: *t*(64) = 5.598, *p <* .001), as did adding the End calibration: Beg+Mid and Beg+Mid+End: *t*(64) = 4.101, *p <* .001, Beg and Beg+Mid+End: *t*(64) = 6.135, *p <* .001. Planned *t*-tests on model type showed FAZE to be significantly better than the other two models (MPIIGaze and FAZE: *t*(64) = 6.542, *p <* .001; ETHXGaze and FAZE: *t*(64) = 3.373, *p* = .001). No differences were found between MPIIGaze and ETHXGaze, *t*(64) = 0.107, *p* = .915.

## Effect of recalibration

We compared the previous best resulting strategy of using Beg+Mid+End calibration blocks with a block-specific (Blockwise) strategy in which single calibration blocks were temporally assigned to the following task block (See Fig.1, B1). That is, the beginning calibration sample was used to predict fixation performance in the first task block, and the middle calibration for the second block. A two-factor ANOVA showed significant main effects of model type, *F*(2, 128) = 9.720, *p <* .001, η^2^ = 0.112, and calibration strategy, *F*(1, 64) = 78.134, *p <* .001, η^2^ = 0.047, and no interaction effects, *F*(2, 128) = 0.300, *p* = .742, η^2^ < 0.001. FAZE predictions were significantly better than the other two models (FAZE and MPIIGaze: *t*(64) = 6.777, *p <* .001); FAZE and ETHXGaze: *t*(64) = 3.570, *p* = .001 while there was no difference between MPIIGaze and ETHXGaze, *t*(64) = 0.009, *p* = .992. Fixation accuracy was significantly higher when using Beg+Mid+End calibration strategy, *t*(64) = 8.839, *p <* .001.

We also compared Blockwise strategy with the calibration performance when using single calibration blocks (Beg, Mid and End) to predict all fixation trials–ensuring equal calibration data size in all cases. Mid calibration block yielded the best results followed by Blockwise, Beg and then the End calibration blocks (see Fig.1, B2); however, the differences were not significant.

## Effect of calibration type

We proceeded with the Beg+Mid+End calibration sample and checked for the effect of calibration task (fix-point calibration on the letter E: E, and smooth pursuit: SP). Fixation trials were evaluated using calibration data from the two calibration tasks separately (E, SP) and pooled (E+SP). We analyzed the data again by a two-factor ANOVA, with model (MPIIGaze, FAZE, and ETHXGaze) as one factor and calibration strategy (E, SP, E+SP) as the other. The main effect of calibration strategy was significant, *F*(2, 128) = 15.716, *p <* .001, η^2^ = 0.006, and so was that of model, *F*(2, 128) = 11.886, *p <* .001, η^2^ = 0.146. The factors did not interact, *F*(4, 256) = 2.311, *p* = .06, η^2^ = 0.001. Once again, FAZE was significantly better than the other two models (FAZE and MPIIGaze: *t*(64) = 9.26, *p <* .001; FAZE and ETHXGaze: *t*(64) = 3.473, *p <* .001), with no difference between MPIIGaze and ETHXGaze, *t*(64) = 0.380, *p* = .705. Planned comparisons showed a significant difference between E and E+SP, *t*(64) = 6.215, *p <* .001, as well as between SP and E+SP, *t*(64) = 4.622, *p <* .001, meaning the combined E+SP strategy was better than either strategy alone. There was no significant difference between E and SP calibration, *t*(64) = 1.945, *p* = .056. Results are plotted in Fig.1C.

The three analyses above showed a clear preference for a larger calibration sample size in all conditions; therefore, we further explored the effect of sample size on individual calibration performance (see Supplementary Fig. 2). Calibration data sample size had only a weak negative correlation with average gaze error, *r*(63) = -.28, *p* = .02. Moreover, the best and worst fixation accuracies were from different subjects for each gaze prediction model, highlighting that additional features (e.g., their head pose or environment) are influential in the final gaze prediction accuracy of models and that these features affect each model’s prediction differently.


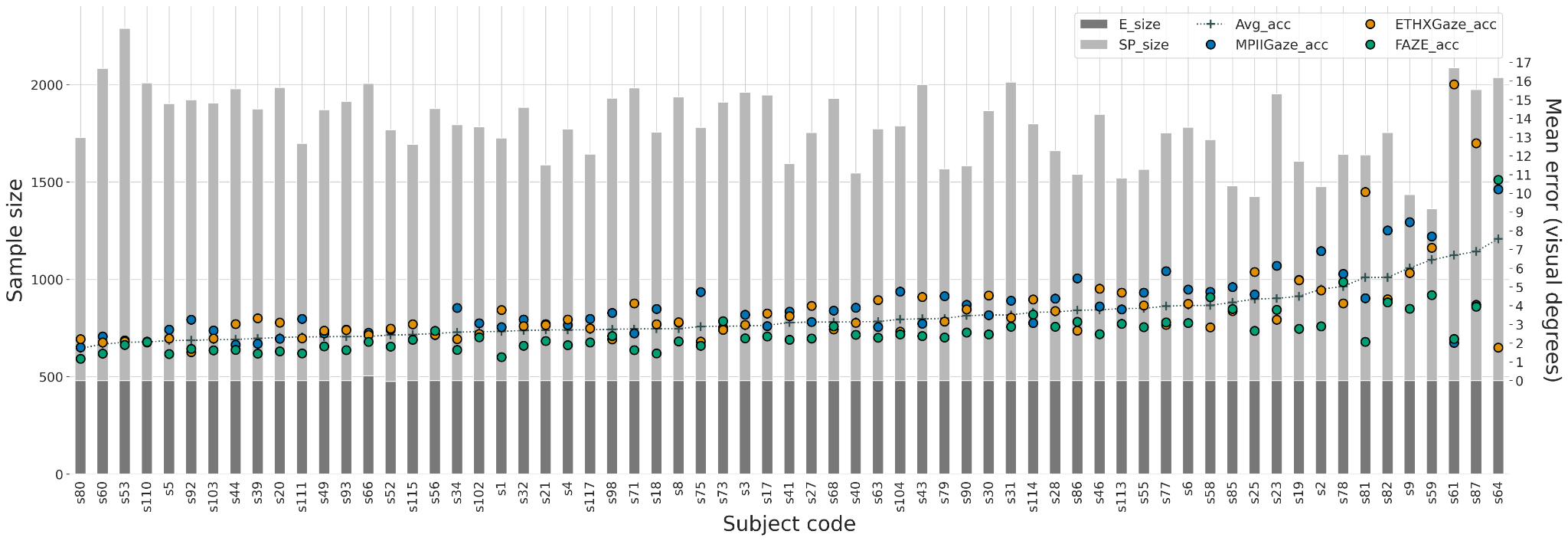


**Supplementary Fig.2**. Subject-wise count of Beg+Mid+End calibration sample size for the three conditions: fix-point (E) only (dark gray), smooth pursuit (SP) only (light gray) and pooled E+SP. The dots represent fixation task errors from the three models’ predictions, with the line representing their average for each subject in the E+SP condition. Participants are sorted left to right, from fewest to most degrees of visual error, averaged across all three models.
